# HPV Strain Predicts Severity of Juvenile-Onset Recurrent Respiratory Papillomatosis with Implications for Disease Screening

**DOI:** 10.3390/cancers13112556

**Published:** 2021-05-23

**Authors:** Mary C. Bedard, Alessandro de Alarcon, Yann-Fuu Kou, David Lee, Alexandra Sestito, Angela L. Duggins, Marion Brusadelli, Adam Lane, Kathryn A. Wikenheiser-Brokamp, Susanne I. Wells, David F. Smith

**Affiliations:** 1 Division of Oncology, Cincinnati Children’s Hospital Medical Center, Cincinnati, OH 45229, USA; mary.bedard@cchmc.org; 2 Division of Pediatric Otolaryngology–Head and Neck Surgery, Cincinnati Children’s Hospital Medical Center, Cincinnati, OH 45229, USA; Alessandro.deAlarcon@cchmc.org (A.d.A.); Yann-Fuu.Kou@cchmc.org (Y.-F.K.); Alexandra.Sestito@cchmc.org (A.S.); Angie.Duggins@cchmc.org (A.L.D.); 3 Department of Otolaryngology–Head and Neck Surgery, University of Cincinnati College of Medicine, Cincinnati, OH 45229, USA; 4 Division of Pediatric Otolaryngology—Head and Neck Surgery, Children’s Hospital of Philadelphia, Philadelphia, PA 19104, USA; leedr@email.chop.edu; 5 Division of Clinical Operations, Medpace, Cincinnati, OH 45227, USA; M.brusadelli@medpace.com; 6 Department of Pediatrics, University of Cincinnati College of Medicine, Cincinnati, OH 45267, USA; Adam.Lane@cchmc.org; 7 Division of Bone Marrow Transplantation and Immune Deficiency, Cincinnati Children’s Hospital Medical Center, Cincinnati, OH 45229, USA; 8 Division of Pathology & Laboratory Medicine and The Perinatal Institute Division of Pulmonary Biology, Cincinnati Children’s Hospital Medical Center, Cincinnati, OH 45229, USA; Kathryn.Wikenheiser-Brokamp@cchmc.org; 9 Department of Pathology & Laboratory Medicine, University of Cincinnati College of Medicine, Cincinnati, OH 45267, USA; 10 Division of Pulmonary and Sleep Medicine, Cincinnati Children’s Hospital Medical Center, 3333 Burnet Ave. MLC 2018, Cincinnati, OH 45229, USA; 11 Center for Circadian Medicine, Cincinnati Children’s Hospital Medical Center, 3333 Burnet Ave. MLC 2018, Cincinnati, OH 45229, USA

**Keywords:** juvenile-onset recurrent respiratory papillomatosis, JoRRP, RRP, laryngeal papillomas, low-risk HPV, HPV strain

## Abstract

**Simple Summary:**

Although it is known that low-risk HPV causes juvenile-onset recurrent respiratory papillomatosis (JoRRP), viral factors such as HPV strain are not routinely determined as part of clinical care. Age at disease onset and HPV strain are two commonly discussed potential predictors of increased disease severity; however, research has been inconclusive and has not yet impacted clinical diagnosis or management of patients with JoRRP. In this prospective study, we found that HPV11+ disease was associated with earlier age of onset, need for surgical intervention, and higher risk of progression to malignancy. These results support that determination of HPV6+ vs. 11+ disease status represents a predictive screening biomarker for disease severity and progression in JoRRP patients. To this end, we provide proof of concept that demonstrates the utility and feasibility of HPV genotyping using RNA-ISH for screening of HPV11+ disease.

**Abstract:**

Juvenile-onset recurrent respiratory papillomatosis (JoRRP) is the most common benign neoplasm of the larynx in children, presenting with significant variation in clinical course and potential for progression to malignancy. Since JoRRP is driven by human papillomavirus (HPV), we evaluated viral factors in a prospective cohort to identify predictive factors of disease severity. Twenty children with JoRRP undergoing routine debridement of papillomas were recruited and followed for ≥1 year. Demographical features, clinical severity scores, and surgeries over time were tabulated. Biopsies were used to establish a tissue bank and primary cell cultures for HPV6 vs. HPV11 genotyping and evaluation of viral gene expression. We found that patients with HPV11+ disease had an earlier age at disease onset, higher frequency of surgeries, increased number of lifetime surgeries, and were more likely to progress to malignancy. However, the amplitude of viral E6/E7 gene expression did not account for increased disease severity in HPV11+ patients. Determination of HPV strain is not routinely performed in the standard of care for JoRRP patients; we demonstrate the utility and feasibility of HPV genotyping using RNA-ISH for screening of HPV11+ disease as a biomarker for disease severity and progression in JoRRP patients.

## 1. Introduction

Recurrent respiratory papillomatosis (RRP), characterized by papillomas of the aerodigestive tract, is caused by the human papilloma virus (HPV) low-risk strains HPV6 and HPV11. RRP is the most common benign neoplasm of the larynx in children [1], with an estimated incidence in the US of 4.3 per 100,000 [2,3]. Particularly in juvenile-onset RRP (JoRRP), nearly all patients are positive for HPV, with HPV6 positivity being more common than HPV11 [4]. Patients typically present with voice changes, stridor, hoarseness and chronic cough, but more serious recurrent pneumonias and acute respiratory distress can also occur [1]. In 3–5% of pediatric cases, papillomas can extend below the larynx and involve the trachea and main stem bronchi [5]. Mortality is significantly higher in roughly 1% of patients with lung parenchymal involvement as compared to patients with a localized laryngeal disease [6,7], particularly as progression to other sites in the aerodigestive tract carries an increased risk of malignant conversion [8]. Importantly, the clinical course of RRP is highly variable without existing predictive biomarkers for patients at high risk for disease progression. 

The most common therapy for children with laryngeal papillomas is surgical debulking [5]. RRP can be a persistent disease, often requiring multiple surgical excisions. The timing of surgical interventions is determined by clinical judgment and patient symptoms. Indications for additional adjuvant medical therapy include the presence of papillomas outside of the larynx or the need for surgical excision more than four times per year [1]. Adjuvant medical therapy is required in as many as 20% of RRP cases [9]. The existing array of adjuvant therapies demonstrate variable efficacy, but no single treatment is uniformly effective at eliminating RRP in pediatric patients [10]. For this reason, recent work has focused on individualizing therapy based on RRP-specific alterations [5,11].

Several studies have sought to identify RRP features predictive of clinical severity, with inconsistent results. Numerous case studies of HPV11+ RRP patients have been reported that detail the fatal progression of the disease [12,13,14,15,16,17,18]. Similarly, analysis of retrospective cohorts has suggested that HPV11+ disease is associated with worse outcomes [19,20,21,22,23,24]. However, others have countered these findings [25,26,27,28] or concluded that an early age of onset, and not the HPV strain, predicts disease severity [29,30,31,32]. These contradictory conclusions have precluded screening for HPV11 as part of an accepted standard of clinical care. Additionally, although RNA-ISH was recently used to evaluate the amplitude of viral gene expression in RRP patient biopsies for the first time [33], it remains unknown whether the distribution and amplitude of viral genes in the HPV11+ disease specifically vary from HPV6 disease.

Currently, testing and screening for HPV6 versus HPV11 are not performed as part of the clinical standard of care for JoRRP. The lack of HPV strain-specific testing not only limits potential clinical guidance but additionally hinders discovery research using human specimens in determining the clinical impact of HPV6 vs. HPV11. Since HPV strain testing for HPV6+ vs. HPV11+ disease is not routinely conducted in clinical practice during diagnosis and management of RRP patients, this creates additional challenges in analyzing retrospective cohorts where tissue may not be available for analysis. In this study, we prospectively recruited a cohort of JoRRP patients in order to evaluate demographic and viral features, including HPV6 vs. 11 status and viral gene expression, as predictors of clinical outcomes, and to stress the importance of identifying HPV11+ patients for increased surveillance and clinical monitoring for disease progression. We find that HPV11+ JoRRP patient status is associated with measures of increased disease severity, leading to the recommendation of strong consideration for HPV genotyping as a screening tool in JoRRP patients. Importantly, we present a workflow using HPV6 or 11 strain-selective E6/E7 targeted RNA-ISH probes that show promise in proof-of-concept studies.

## 2. Materials and Methods

### 2.1. Study Participants

Patients 1 to 17 years of age with a history of RRP met inclusion criteria for participation in the study. Basic demographic data, such as age at onset, sex, and race were recorded, along with the number of prior surgeries at the time of recruitment. Recorded laryngoscopy videos were used to determine the Derkay score, a validated clinical staging system that includes both a subjective assessment of clinical parameters and an anatomic assessment of disease distribution by subsite of the aerodigestive tract [34,35].

### 2.2. Biopsy of RRP Lesions and Primary Cell Culture

Biopsies were obtained and used to establish primary mucosal squamous epithelial cell cultures as previously described [36]. Importantly, matched biopsy sets of papilloma and non-diseased tissue were obtained per patient. Papillomas were taken from the larynx, isolated to the supraglottis, glottis, or subglottis, and non-diseased tissue from the posterolateral arytenoid mucosa where no gross disease was present. 

### 2.3. HPV Genotyping

2D cell pellets were collected from early passage (passage 2–4) primary cells derived from papilloma biopsies, extracted for RNA, and run by RT-qPCR using HPV-strain specific primers to determine HPV6 versus HPV11 genotype as previously described [36].

### 2.4. Fluorescent RNA In Situ Hybridization (RNA-ISH) for HPV Gene Expression

mRNA expression levels were detected using the RNAscope^®^ 2.0 Manual Assay and joint HPV6/11 probe for E6/E7 per manufacturer’s instructions (Advanced Cell Diagnostics, Inc., Newark, CA 415211, USA). In brief, paraffin-embedded biopsies were sectioned at 5 µm thickness onto acid-treated SuperFrost Plus glass slides and baked overnight at 37 °C. RNA in situ hybridization followed the standard protocol for FFPE tissue sections with pretreatment for the RNAscope^®^ 2.5 Assay. Cell membranes were stained for visualization of tissue using wheat germ agglutinin (Thermo Fisher, Waltham, MA, USA W849) or by immunofluorescence for E-Cadherin. Briefly, tissue was blocked with 10% donkey serum (Jackson Immunoresearch Laboratories, West Grove, PA, USA 017-000-121) in PBS for 1 h, incubated in E-Cadherin antibody (R&D Systems, Minneapolis, MN, USA AF648) in antibody dilution buffer (3% BSA, 0.5% Triton-X100, 0.05% Tween-20, 0.04% sodium azide in PBS) and 2% donkey serum for 1 hr at room temperature, washed, and incubated in secondary antibody (Jackson Immunoresearch Laboratories, West Grove, PA, USA 715-295-150) diluted in antibody dilution buffer with 0.5 µL 4′,6-diamidino-2-phenylindole (DAPI) (Thermo Fisher, Waltham, MA, USA D1306). The sections were mounted in ProLong Gold mountant (Thermo Fisher, Waltham, MA, USA P36934) and visualized by confocal microscopy.

### 2.5. Statistical Methods

Negative binomial regression was computed with R software version 4.0.0 (The R Foundation, Vienna, Austria), all other statistics were performed using GraphPad Prism6 software (version 9.0.0, GraphPad Software, San Diego, CA USA). The targeted sample size for comparisons was 20 participants. At this sample size, there is a greater than 90% power to detect an effect size (absolute difference in RRP vs. N/corresponding standard deviation) equal to or greater than 0.8 at the 0.05 level of significance. To analyze risk factors for the number of lifetime surgeries at the end of the study period, a negative binomial regression with an offset of log(time post-diagnosis) was used to calculate the model estimated incidence rate ratio (IRR). Time at risk for surgical interventions was defined as the amount of time (in years) since a patient was diagnosed with RRP, during which surgeries could occur as part of clinical management. When *p* ≤ 0.05, an IRR > 1 indicated that group 1 had a greater risk than group 2 and vice versa. Race, sex, and HPV strain were calculated as categorical variables, and age at onset and Derkay score as continuous variables. Numerical data is summarized by the average, standard deviation (SD), and range. Statistical significance between unpaired groups was determined by Kruskal-Wallis across categories or the nonparametric Mann-Whitney test to account for small sample size and non-normal distribution.

### 2.6. DNA In Situ Hybridization (DNA-ISH) for Research

For DNA-ISH images in Figure 4, FFPE tissue sections were stained manually using a modified protocol as follows. Slides were deparaffinized and a peroxidase block was performed using 0.3% hydrogen peroxide for 5 min at room temperature (RT) prior to antigen retrieval in 1× Diva Decloacker reagent (Biocare Medical, Pacheco, CA, USA DV2004MX) for 40 min in a Biocare Decloaking chamber pre-heated to 40 °C. Slides were cooled for 10 min, washed in diH_2_O, and lightly dried to draw hydrophobic barriers (Vector laboratories, Burlingame, CA, USA H-4000). Pronase was diluted 1:30 in Pronase buffer and applied to slides for 10 min at RT (Biocare Medical, Pacheco, CA, USA PRT957). Slides were washed in diH_2_O and placed in an opaque humidity chamber. Probes (HPV6, Biocare Medical, Pacheco, CA, USA BRA4045; HPV11, Biocare Medical, Pacheco, CA, USA BRA4046) were selected per patient to match HPV strain determined previously by RT-qPCR. Probes were diluted in a DNA hybridization buffer (Biocare Medical, Pacheco, CA, USA BRI4036G10) to a concentration of 0.10 ng/uL, applied to tissue, coverslipped with 12 mm glass circles (Fisher Scientific, Hampton, NH, USA 12-545-80), de-natured for 5 min at 95 °C, and hybridized overnight at 40 °C. Slides were washed in a 2× SCC buffer for 5 min at 40 °C then in 1× TBS for 3 min at RT. Mouse anti-Digoxigenin (Biocare Medical, Pacheco, CA, USA BRI4049G) was applied for 15 min at RT, then slides were washed with TBS. MACH2 Mouse HRP (Biocare Medical, Pacheco, CA, USA MHRP520H) was applied for 30 min at RT, slides were washed with TBS, stained with DAB (Vector laboratories, Burlingame, CA, USA SK-4100) per manufacturer instructions, counterstained with hematoxylin and bluing reagent, and coverslipped with toluene.

### 2.7. DNA-ISH Assay for Screening Low-Risk HPV6/11

In situ hybridization (ISH) was performed on a Ventana Discovery Ultra automated staining platform (Roche Tissue Diagnostics, Indianapolis, IN, USA 5870305001). Staining for Low Risk (Type 6/11) HPV was performed using the DIGX HPV Type 6/11 Probe (Enzo Life Sciences, Farmingdale, NY, USA ENZ-GEN112) against type 6 and 11 DNA, using a laboratory-developed assay. Slides were deparaffinized online and pretreated with Protease 3 (Roche Tissue Diagnostics, Indianapolis, IN, USA 760-2020) for 16 min at 37 °C. Slides were then denatured at 90 °C for 4 min. The DIGX HPV6/11 probe was diluted 1:1 using an In Situ Hybridization buffer (Enzo Life Sciences, Farmingdale, NY, USA ENZ-33808) before application and hybridization were performed at 45 °C for 2 h. Stringency washing was performed through a gradient of SSC washes at 55 °C. Biotinylated anti-DIG antibody (Sigma-Aldrich, St. Louis, MO, USA B7405) was applied for 20 min at 37 °C, and then detected with the BlueMap alkaline phosphatase detection kit (Roche Tissue Diagnostics, Indianapolis, IN, USA 760-120). Slides were counterstained with nuclear fast red (Poly Scientific R&D, Bay Shore, NY, USA S248) and permanently mounted.

### 2.8. RNA-ISH Assay for Screening HPV6 vs. HPV11

Staining for type 6 and 11 E6/E7 mRNA was performed using Ventana HPV 6 mRNA and HPV 11 mRNA probes (Roche Tissue Diagnostics, Indianapolis, IN, USA; HPV6, 760-1239 and HPV11, 760-1240) using a laboratory-developed assay on a Ventana Discovery Ultra automated staining platform (Roche Tissue Diagnostics, Indianapolis, IN, USA 5870305001). Slides were deparaffinized online and pretreated with Protease 3 (Roche Tissue Diagnostics, Indianapolis, IN, USA 760-2020) for 16 min at 37 °C followed by cell conditioning with CC2 (Roche Tissue Diagnostics, Indianapolis, IN, USA 950-223) at 90 °C for an 8 min cycle. Slides were then denatured at 90 °C for 4 min and hybridization was performed at 45 °C for 4 h. Stringency washing was performed through a gradient of SSC washes at 55 °C. Biotinylated anti-DNP antibody (Invitrogen, Carlsbad, CA, USA A-6435) was applied for 20 min at 37 °C, and then detected with the BlueMap alkaline phosphatase detection kit (Roche Tissue Diagnostics, Indianapolis, IN, USA 760-120). Slides were counterstained with nuclear fast red (Poly Scientific R&D, Bay Shore, NY, USA S248) and permanently mounted.

## 3. Results

### 3.1. Summary of the Published Literature on Risk Factors of Severe RRP Disease

Risk factors in RRP were identified in published cohorts through literature review, and the results are summarized in Table 1. The results highlight substantial controversy in the field regarding the role of HPV11 in an aggressive clinical course.

### 3.2. Cohort Recruitment and Characteristics

**Figure 1:**
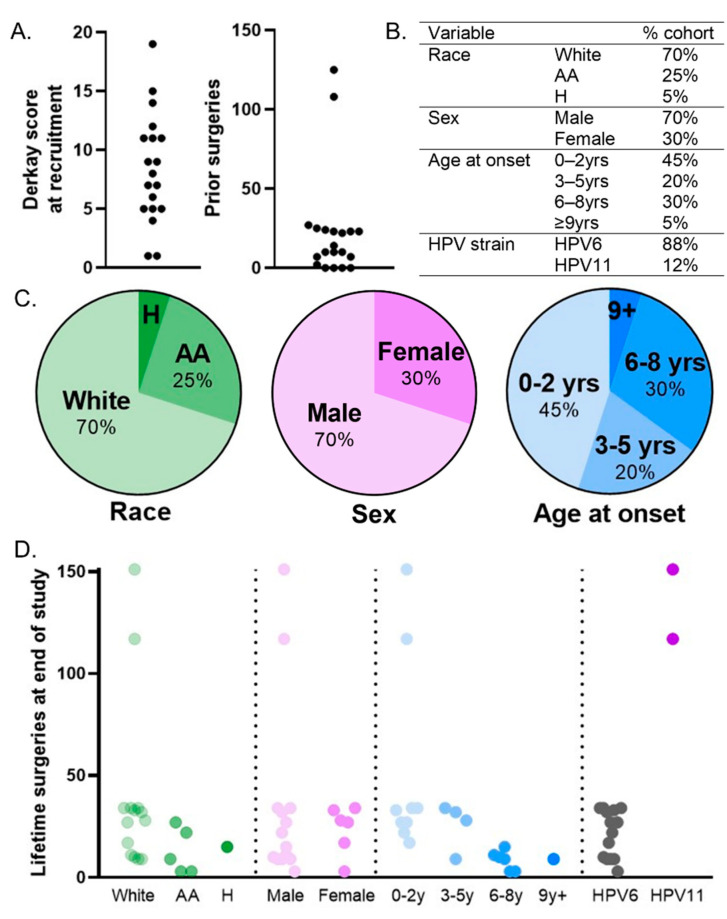
Clinical and demographical characteristics of the recruited JoRRP cohort. (**A**) At the time of recruitment, measures of disease severity across 20 patients in the cohort showed an average Derkay score of 8.0 (range 0–19) and 23 previous surgeries (range 0–125). (**B**) Summary of demographical characteristics of the cohort from Table 2. (**C**) Patient characteristic summary graphs indicating that patients were predominately white and male and had an early (≤5 yrs) age at disease onset. (**D**) The number of lifetime surgeries at the end of the study is broken down by demographical sub-categories. H = Hispanic, AA = African American.

Twenty study participants were recruited to the cohort from July 2015–July 2019 and followed for a minimum of one year. Children included in the study were previously diagnosed with RRP and presented to Cincinnati Children’s Hospital Medical Center (CCHMC) for surgical consultation. First, we sought to determine patient characteristics and tabulate main outcomes and measures (Table 2). The Derkay score, a validated clinical staging system [34,35], was determined from recorded laryngoscopy videos (range 0–19, average 8.0). The Derkay score includes both a subjective assessment of clinical parameters as well as an anatomic assessment of disease distribution by the subsite of the larynx, trachea, and respiratory tract. The number of surgeries prior to recruitment (range 0–125, average 23) was assessed (Table 2). Derkay scores and the number of surgical interventions (Figure 1A) were used as indications of clinical severity within the patient cohort.

At the end of the study period, we calculated the number of surgeries within the first year of recruitment (range 1–8, 4.4 ± 2.4), lifetime surgeries calculated after one year of enrollment (range 3–131, average 27), and up to the end of the study period (range 3–151, average 31) (Table 3). Finally, all patients were reported to be low-risk HPV+ in our chart review; however, we sought to determine the specific HPV strain (HPV6 vs. 11) for each patient. To this end, fresh papillomatous (RRP) and adjacent uninvolved control (non-diseased) tissue collected from each patient was cultured to establish patient-derived keratinocyte cell populations that represent the target cells and reservoirs of HPV infection. Successful keratinocyte cultures were obtained for 80% (16/20) of patients, with the remaining four patients having insufficient tissue and/or lack of need for repeat surgeries to obtain additional tissue for culture. Cell pellets from these cultures were analyzed by RT-qPCR using HPV6- and HPV11-specific primers. No RRP-derived cells tested positive for both HPV6 and 11. The data are compiled in Table 3. 

We analyzed the proportion of the cohort in sub-categories for the race, sex, age at onset, and HPV strain (Figure 1B). We found that the patient cohort was predominantly white (70%) and male (70%) (Figure 1C). Prior studies have found early age at disease onset, specifically 2 years of age or younger, to be a predictive factor of disease severity and/or sequela [3]. For this reason, age at onset was binned by every 3 years and showed that nearly half (45%) had an age at onset of 2 years or younger (Figure 1C). In accordance with the literature, 88% (14/16 tested) of the patient papillomas were HPV6+, with two patient samples testing positive for HPV11 allowing for HPV genotype comparisons in the patient cohort. The number of lifetime surgeries at the end of the study was graphed for the sub-categories of each variable (Figure 1D). However, the number of lifetime surgeries a patient has had thus far is also dependent on the number of years since the diagnosis with JoRRP. We noted that the time during which patients were at risk, or eligible, for surgical interventions (equal to the time interval since diagnosis) was highly variable (0–14 years) and was important to take into account for comparisons.

### 3.3. Analysis of Predictive Factors Reveals Importance of HPV Type

**Figure 2:**
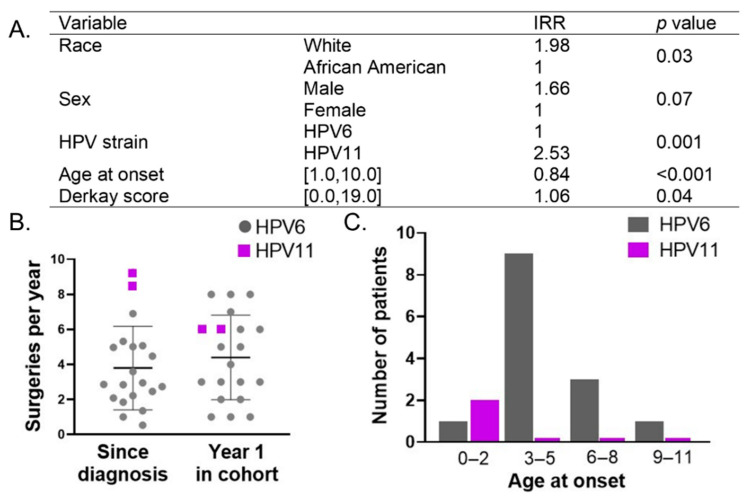
HPV11+ versus HPV6+ status, and not the age of onset, is associated with worse clinical course and increased severity. (**A**) Results of negative binomial regression analysis on lifetime surgeries at the end of the study period shows that white patients, males, HPV11+ patients, and patients with high Derkay scores had an increased risk of high surgical interventions, whereas patients with a later age of onset had a decreased risk of the need for surgical interventions. (**B**) The average number of surgeries per year since diagnosis and during the first year after recruitment for HPV6+ (gray) and HPV11+ (purple) patients. HPV11+ patients had increased surgeries per year since diagnosis (*p* = 0.02) but not in the first year after recruitment (*p* = 0.58). (**C**) Age at disease onset distribution HPV6+ and 11+ patients show an association between HPV11 positivity and earlier age at disease onset (*p* = 0.04).

We tested whether race, sex, age at onset, Derkay score, and/or HPV strain were predictive of disease severity, as assessed by the number of lifetime surgeries at the end of the study period. Negative binomial regression was used in order to account for differences in time at risk for surgeries (time since diagnosis), where an estimated incidence ratio (IRR) greater than 1 indicated greater risk between groups. We found that white patients, males, patients with higher Derkay scores, and HPV11+ patients had an increased risk of surgical interventions and thus disease severity, whereas patients with an older age at onset had a reduced risk of severe disease (Figure 2A). We additionally analyzed disease severity over time by calculating the frequency of surgeries per year since diagnosis and the number of surgeries in the first year of the prospective study. In the first year of recruitment, patients underwent an average of 4 surgeries, (SD 2, range 1–8), which approximated the average surgeries per year since disease diagnosis to be 4 (SD 2, range 1 to 9) (Figure 2B). Notably, patients clustered around either a higher frequency (>4 surgeries/year or every ≤10 weeks) or lower frequency (≤4 surgeries/year or every three months or less) of surgical interventions. HPV11+ patients had an increased number of surgeries per year since diagnosis (*p* = 0.02) as compared to HPV6+ patients that were not evident in the first year of recruitment (*p* = 0.58) (Figure 2B). This suggests that the clinical severity of HPV11+ disease is due to consistently high numbers of surgeries required during the entire disease duration rather than more aggressive disease during an individual year. In contrast to the previous negative binomial regression analysis, analyzing the frequency of surgical interventions per year does not take into account the variable time since diagnosis across patients, thus additional prospective information is required to confirm this finding. Importantly, HPV11+ patients had an earlier age at onset than HPV6+ patients (*p* = 0.04, Figure 2C). Analysis of our cohort supports younger age at disease onset as a predictor for increased need for surgical interventions. The data also highlight features associated with the HPV11+ disease, namely younger age at disease onset and a higher need for frequent surgeries, which underlies an overall increased risk for lifetime surgical interventions. 

### 3.4. Clinical Course Categories Show Differences in Age of Onset and Disease Severity

**Figure 3:**
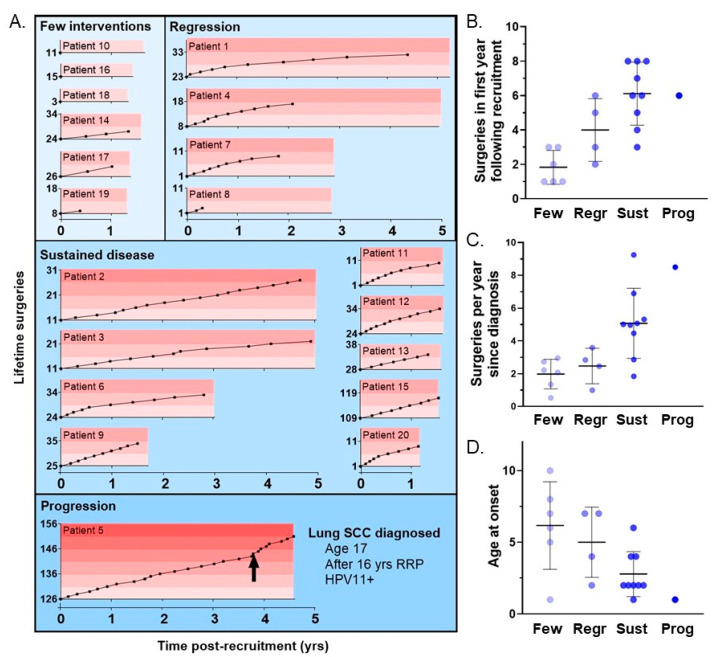
Patient clinical course categorized by low surgical frequency, sustained disease, disease regression, or disease progression (**A**) Clinical course per patient with the y-axis starting at the surgical number at the time of recruitment. Patients were categorized by few surgical interventions (defined as ≤2/year), disease regression (defined as a decrease to ≤1/year), sustained disease, or disease progression (diagnosis of lung SCC indicated by arrow). Across the clinical course groups, the number of surgeries in the first year following recruitment (**B**), the average surgeries per year since disease onset (**C**) and the age at disease onset (**D**) show statistically significant differences across the groups (*p* < 0.001, *p* = 0.04, and *p* = 0.02, respectively).

Over the course of the study, patients were categorized into different groups based upon the need for few surgical interventions (6 patients, defined as ≤2 per year), disease regression (4 patients, defined as a decrease to ≤1 per year), sustained disease (9 patients), or disease progression to lung squamous cell carcinoma (SCC, 1 of 2 HPV11+ patients) (Figure 3A). Kruskal-Wallis Test was conducted to examine differences across the assigned clinical course categories. No significant differences were found for years of the disease since diagnosis (*p* = 0.54), Derkay score at recruitment (0.72), or surgeries prior to recruitment (0.12) (Table 4). As expected, a significant difference among the four categories was found in the number of surgeries in the first year after recruitment (*p* < 0.001, Figure 3B). Additionally, the average surgeries per year since diagnosis (*p* = 0.004, Figure 3C), and total lifetime surgeries (*p* = 0.04) were also statistically significant across categories. Consistent with the prior analysis that shows the correlation between earlier age at onset and increased risk of surgical interventions, a significant difference in age at onset across categories was noted (*p* = 0.04, Figure 3D). Age at onset in the few surgical interventions and sustained disease groups was directly compared demonstrating a statistically significant difference (*p* = 0.04). This indicated that patients with an earlier age of onset were more likely to fall into the category of sustained disease.

### 3.5. Viral Gene Expression Levels Do Not Account for Disease Severity Differences between HPV6 and HPV 11 Cohorts 

**Figure 4:**
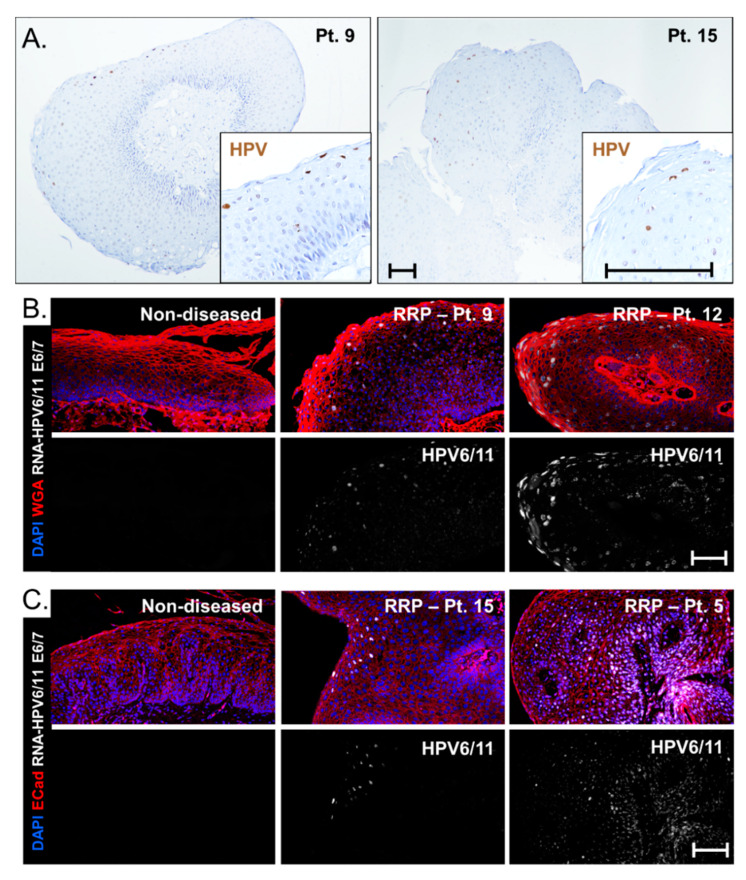
HPV viral gene expression levels vary across patients independent of HPV genotype. (**A**) Routine DNA-ISH on papillomas shows HPV+ positive cells predominantly in the more superficial layers of the epithelium. Scale bar, 100 µm. HPV6/11 viral E6 and E7 gene expression (white) by RNA-ISH is variable in both (**B**) HPV6+ and (**C**) HPV11+ patient samples; scale bar, 100 µm. Two patient samples are shown for both HPV6+ and HPV11+ samples. Nuclei are highlighted by DAPI stain (blue) with cell membranes stained in red by WGA (**B**) or ECad (**C**).

DNA-ISH is routinely clinically performed on FFPE biopsies at some centers to confirm the presence of low-risk HPV strains (representative images, Figure 4A), but this assay does not indicate RNA expression levels. To determine whether the extent of HPV viral gene expression could underlie the association between HPV strain and disease severity, RNA-ISH using a cross-reactive HPV6/11 probe against E6 and E7 was implemented. Patients were classified by HPV6+ (Figure 4B) and HPV11+ (Figure 4C) based on RT-qPCR data. No signal was detected in non-diseased tissue controls. Levels of HPV gene expression in papillomas varied significantly in both HPV6 and HPV11 patients; low levels of viral gene expression were seen in patient 9 (HPV6+) and patient 15 (HPV11+) and high levels were detected in patient 12 (HPV6+) and patient 5 (HPV11+). Thus, HPV11+ disease does not consistently result in increased viral E6/E7 gene expression as compared to HPV6 disease. Interestingly, the HPV11+ patient that progressed to lung SCC had strong HPV E6/E7 expression in basal cells as compared to suprabasal cells. This cell-selective expression was not observed in specimens from other patients and may indicate integration of HPV11 into the cellular genome of this patient, a known risk factor for HPV-associated carcinogenesis [38]. 

### 3.6. HPV Strain-Selective Probes for RNA-ISH to Enable HPV6 versus HPV11 Screening

**Figure 5:**
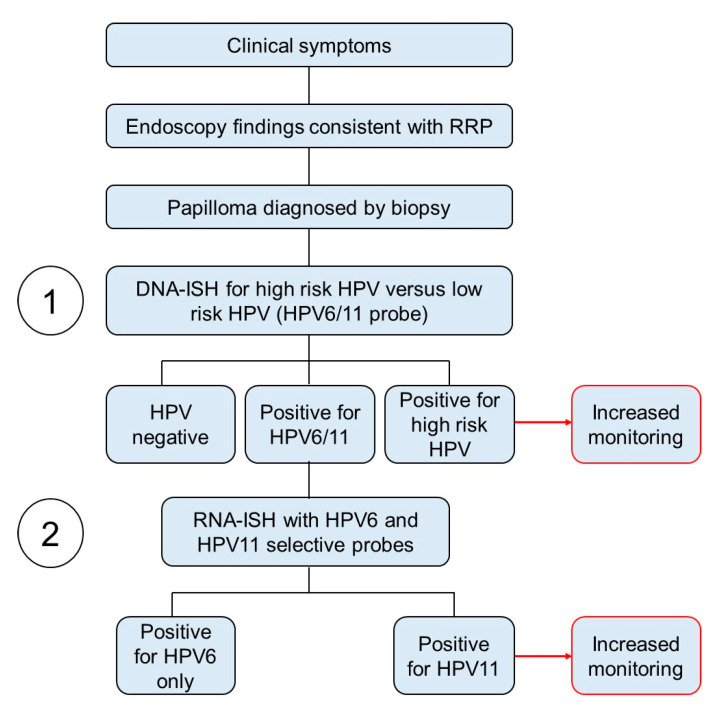
Proposed pipeline for screening HPV11. Clinical symptoms determine the need for endoscopy with biopsy being performed when masses are visualized. Pathologic diagnosis of papilloma results in routine clinical testing in most centers to determine low-risk versus high-risk HPV status (Step 1). A recommendation is proposed to supplement this initial step by testing those biopsies positive for low-risk HPV using HPV strain-selective probes (Step 2) in order to identify HPV11 positive patients that should be more aggressively monitored for disease progression and need for treatment intervention (emphasized in red).

In order to facilitate screening for HPV11, we explored the feasibility of using commercially available HPV strain-selective RNA E6/E7 targeted ISH probes on patient tissue samples from our patient cohort in a proof-of-concept study. Currently, patients are screened for low-risk versus high-risk HPV status by performing ISH on tissue samples using combined DNA-ISH probes that detect multiple different low-risk or high-risk HPV subtypes, respectively. We concurrently tested papilloma specimens from our patient cohort for the presence of low-risk HPV using a commercially available combined DNA-ISH probe along with testing for HPV6 and HPV11 using subtype-selective, E6/E7 targeting, commercially available RNA-ISH probes (Figure 5). The assay protocols were prioritized for sensitivity with the combined low-risk HPV DNA probe and to eliminate cross-reactivity with the HPV6 and HPV11 selective RNA-ISH probes. The known HPV strains of our patients determined by PCR analyses allowed a comparison of the tissue ISH assay results to molecular HPV6 vs. HPV11 strain results.

**Figure 6:**
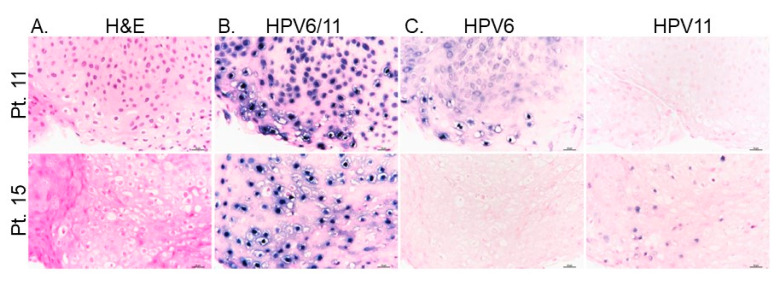
A two-step approach to optimize detection of HPV infection with HPV6/HPV11 subclassification. (**A**) Representative H&Es of papillomas from patient 11 and patient 15 that were previously HPV genotyped as HPV6+ and HPV11+, respectively. (**B**) Clinically validated low-risk combined HPV6/11 DNA-ISH showing robust staining in many cells. (**C**) RNA-ISH using strain-selective HPV6 and HPV11 probes showing restricted HPV6 or HPV11 staining in patients 11 and 15, respectively. Note the reduced number of positive cells using the HPV6 and HPV11 RNA selective probes as compared to the combined HPV6/11 DNA probes on serial sections from the same papillomas. Scale bar, 25 µm.

HPV6 or HPV11 status was successfully determined in papillomas from four patients selected from the cohort, including samples for HPV6+ (Patients 2, 3 and 11) and HPV11+ (Patient 15) (representative H&Es, Figure 6A). All four papillomas contained many cells robustly staining with the combined HPV6/11 Enzo DNA-ISH probes routinely used clinically at our center (Figure 6B). The Enzo HPV6/11 probe was highly sensitive and gave a high signal across all HPV+ cells, a strength for clinical testing, at the expense of the granularity in relative amplitudes provided by the Biocare HPV6/11 probes in Figure 4A. Importantly, three papillomas were found to positively stain with the HPV6 selective RNA probe, but not the HPV11 probe (Figure 6C). Conversely, the papilloma from patient 15 had positive staining with the HPV11 selective probe only (Figure 6C). In all four cases, the RNA-ISH results matched the HPV genotypes determined by RT-qPCR. Additionally, the number of positive cells in all four cases was consistently higher when using the HPV6/HPV11 DNA probes as compared to the HPV6 and HPV11 selective probes. Thus, the results support the anticipated increased sensitivity of the combined HPV6/HPV11 probes that target viral DNA as compared to the HPV6 and HPV11 selective probes targeting E6/E7. It is therefore expected that some samples with a low viral load may not be detected by the HPV6 and HPV11 probes using these conditions. The chance of successfully subtyping the patient can be improved by testing multiple papilloma samples. Four of the patients we tested had biopsies available from two distinct surgical procedures. In two patients, both papilloma biopsies gave similar results, whereas, in the remaining two patients, one biopsy was positive for HPV6 only while the second biopsy had undetectable levels. Together, these proof-of-concept studies provide evidence for the feasibility of developing clinically validated protocols to both sensitively identify HPV-infected papillomas as well as subclassify the HPV6 versus HPV11 status of the patients by tissue-based studies. The heightened sensitivity of the combined low-risk HPV DNA probes together with the higher stringency protocol needed to obtain specificity with the HPV6 and HPV11 selective probes forms the rationale for the recommendation in the proposed clinical algorithm for a two-step approach to determine the HPV status. This two-step approach takes advantage of the highly sensitive combined low-risk HPV DNA test to maximize identification of HPV infection together with the ability to distinguish HPV6 versus HPV11 subtypes with more specific RNA probes to guide patient care.

## 4. Discussion

Previous studies have sought to identify RRP patient characteristics predictive of clinical course and disease severity, including progression to malignancy. Age at disease onset and HPV strain are two commonly discussed potential predictors of increased disease severity; however, research has been inconclusive and has not yet impacted clinical diagnosis or management of patients with JoRRP. Specifically, it is not widely accepted that HPV11 is a risk factor for more severe JoRRP disease and patients are not routinely screened for HPV6 vs. HPV11. Looking at the study designs of multiple studies, summarized in Table 1, it is readily apparent that when adult-onset patients (AoRRP) are included in the analyzed cohorts it is more likely that HPV11 is not found to be a risk factor. For example, a recent study evaluated over 300 patients with RRP, including both juvenile-onset and adult-onset, and stratified patients based on indolent or aggressive disease using composite scoring [32]. The authors concluded that HPV strain is not associated with clinical course across all patients; however, in the JoRRP patients specifically, HPV11+ disease was found to associate with earlier age of diagnosis and aggressive disease. These results emphasize the possibility that JoRRP and AoRRP represent distinct diseases with differing contributions of HPV status to disease severity. This idea is consistent with other published literature on AoRRP. For example, it has been found that the distribution of HPV strains is not consistent between JoRRP and AoRRP [4,39] and, importantly, AoRRP disease is more frequently HPV negative [4]. Furthermore, HPV negative AoRRP was found to be more severe, with a 50-fold increase in risk for malignant transformation [4]. Altogether, disease drivers beyond HPV strain are believed to be key in adult-onset diseases, such as coinfection with viruses such as HIV, HSV, or CMV, these are detected at far lower rates in young children [2,20,37]. 

In this Cincinnati Children’s Hospital Medical Center study, we recruited a prospective cohort to explore risk factors specifically in JoRRP. We confirmed an association between earlier age at disease onset and increased clinical severity in JoRRP, as measured by increased risk for surgical interventions. When patients were grouped into categories based on the observed clinical course during the study period—few interventions, disease regression, sustained disease, or disease progression—differences in age at disease onset were again found to be statistically significant across the categories. While our data aligns with those presented in other studies highlighting the importance of young age at disease onset as an indicator of a more aggressive disease course, the majority of our cohort presented with disease at a young age (45% at ≤2 years, average age of onset 4.2 years). This suggests that other factors contributed to disease progression. With this in mind, age at onset cannot alone be used as a robust predictive tool for patients with JoRRP.

Importantly, our studies demonstrate that patients with HPV11+ JoRRP had an increased risk of surgical interventions (*p* = 0.02) and, thus, exceedingly high numbers of lifetime surgeries. Furthermore, patient outcomes showed HPV11+ disease was not only associated with earlier age at disease onset (*p* = 0.04), but also more average surgeries per year thereafter (*p* = 0.02) and a higher risk of progression to malignancy (absolute risk 1/2 vs. 0/14). From these results, we propose that HPV11 positivity leads to earlier overall age at onset for JoRRP, persists with higher disease burden resulting in more frequent needs for surgical interventions, maintains a clinical course of sustained disease despite adjuvant medical therapy, and has a higher risk of progression to malignant disease. This premise is supported by case reports in the literature detailing the progression of HPV11+ disease to significant, often fatal, sequelae, [12,13,14,15,16,17,18] and publications noting HPV11 to be associated with worse disease in JoRRP patients [19,20,21,22,23]. 

A recent study applied RNA-ISH to RRP patient biopsies for the first time and found an association between disease severity and amplitude of viral gene expression [33]. This finding begs the question of whether viral genes of HPV11+ disease differ in amplitude and distribution compared to HPV6+ disease. We sought to answer this question by analyzing viral E6/E7 gene expression in both HPV6+ and HPV11+ papillomas. We found that the level of E6/E7 expression level varied significantly regardless of HPV strain. Specifically, HPV11+ patients were not found to have notably higher levels of gene expression. We concluded that increased risk conferred by HPV11 is not purely due to increased viral gene expression, but rather due to functional differences in disease-promoting factors present in HPV11 but not HPV6 strains. Importantly, RNA-ISH emphasized a difference in the distribution of HPV gene expression in Patient 5, the HPV11+ patient that progressed to lung SCC. Normally, the HPV viral life cycle is tied to the differentiation program of the stratified epithelium, with the highest gene expression in the most terminally differentiated layers, consistent with DNA ISH staining of biopsies that we observed in Figure 4A. However, HPV gene expression was altered in patient 5 and showed viral expression primarily in basal cells which may reflect a change in the physical state of the virus such as integration. HPV11 is associated with an increased incidence of integration into the host genome [40], a well-accepted risk factor for malignant transformation [38].

Despite our findings and those of others in the literature that suggest a role for HPV11 in disease severity, HPV strain is not routinely determined to direct clinical care of patients with RRP. Instead, testing relies on a simplified low risk (HPV6/11) versus high risk (HPV16/18) categorization that groups HPV6 and 11 together. This standard of clinical practice creates a challenge in studying HPV strain-dependent RRP disease progression since retrospective studies and cross-institutional studies require access to tissue banks and a means to determine distinct HPV strain status. Additionally, this precludes clinicians from identifying those patients with HPV11+ disease that are likely to lead to a striking number (>100) of lifetime surgeries and concerning the risk of dire sequelae, including lung SCC, that may justify increased vigilance in surveillance and management of RRP patients. It is possible that variants and sub-types of HPV6 and HPV11 may differ in virulence and underlie variation in clinical outcomes caused by each strain [28]. However, more evidence is required to support and translate this possibility to actionable testing, such as is possible by screening for HPV11+ disease. 

In order to enable the screening of HPV11 as a biomarker of severe disease in JoRRP patients, a clinically validated testing approach needs to be developed to sensitively detect HPV infection while providing appropriate specificity to differentiate HPV6 and HPV11 subtypes. We propose a two-step testing strategy to maximize sensitivity for detection of low-risk HPV infection using DNA probes combined with a second test to selectively identify HPV6 and HPV11 subtypes using RNA probes. We used commercially available HPV probes combined with an automated ISH protocol in proof-of-concept studies to show the feasibility of developing such a clinical assay to guide patient management. We propose using clinically applied combined low-risk versus high-risk HPV screening with DNA probes to maximize sensitivity. A second testing step, using strain-selective HPV probes optimized to minimize cross-reactive staining between HPV6 and HPV11 is then applied. This second test allows a lower signal sensitivity to prioritize specificity. As a proof-of-concept, we tested this algorithm on patient samples in the cohort and were able to assign HPV6 or HPV11 status to four patients (3 HPV6+ and 1 HPV11+) that matched the HPV genotypes determined by RT-qPCR. Although this two-step assay needs to be further optimized to become a clinically validated assay given the variability in staining of other routinely processed clinical samples, including some samples with no staining or staining with both HPV6 and HPV11 probes, our proof-of-concept results using research samples provides evidence for the feasibility of designing sensitive and strain selective HPV clinical assays to allow for HPV11 screening and guide clinical care.

This study is limited by the small number of 20 participants in our cohort, although sufficient numbers to perform statistical analyses were achieved. Since RRP is a rare disease, the 4-year time frame that was necessary to recruit sufficient patients translated into variable amounts of prospective data and specimens across patients. Small 2–3 mm biopsies typically obtained in the routine surgical management of JoRRP patients precluded the ability to consistently perform the same uniform tests for all study patients, including determining strain. It is possible that patients will segregate into different clinical course categories with extended follow-up time, for example with regards to disease regression or progression. Similarly, it is important in future studies to evaluate potential additional contributing factors to disease progression such as sporadically administered adjuvant treatments that may alter disease outcomes in individual patients despite the lack of consistent responses to medical therapy in RRP patients. 

We established primary sustained patient-derived keratinocyte cultures that will now enable a wide range of studies, including identifying viral mechanisms driving patient-specific outcomes to guide the rational development of novel diagnostic, prognostic and treatment strategies. While we used these cultures to test for HPV strains, determination of HPV6 versus HPV11 can be done directly on papillomas excised during routine surgical diagnostic biopsies and papilloma excisions, using similar protocols as those used currently for low-risk HPV testing. While combined HPV6/11 ISH probes are routinely used to establish infection with low-risk HPV, strain-selective probes for HPV6 and HPV11 can also be applied as demonstrated by our proof-of-concept studies. Thus, the use of strain-selective RNA-ISH probes represents a feasible test to enable the identification of HPV11+ patients who might be at a particular risk of disease recurrence or progression requiring more vigilant patient management and treatment. 

## 5. Conclusions

HPV11+ RRP was associated with earlier age of onset, heightened need for surgical intervention, and higher risk of progression to malignancy. HPV11+ disease status represents a predictive screening biomarker for disease severity and progression in JoRRP patients. To this end, we propose a clinical testing algorithm incorporating a clinically practical two-step testing process to assess for HPV11 status to guide patient care. 

## Figures and Tables

**Table 1:** Cohorts of RRP evaluating risk factors, in order of publication year.

Study	Cohort Composition *	Method of HPV Genotyping **	Risk Factors for Severe Disease	Is HPV11^+^ Disease More Severe?
Mounts, 1984 [27]	21; AoRRP + JoRRP		HPV6 subtype c	Unknown ^1^
Padayachee, 1993 [26]	20; JoRRP	ISH	HPV6	No
Pou, 1995 [20]	29 (24); AoRRP	PCR	HPV11/16; HSV, EBV, or CMV coinfection	Unknown ^2^
Rimell, 1997 [37]	24; JoRRP	PCR	HPV11	Yes
Gabbott, 1997 [29]	47; JoRRP	PCR	Early age at diagnosis	No
Peñaloza-Plascencia, 2000 [25]	47; JoRRP	PCR	None	No
Rabah, 2001 [22]	61; JoRRP	PCR	HPV11	Yes
Wiatrak, 2004 [19]	73 (58); JoRRP	ISH, PCR	HPV11, age at onset ≤3, Medicaid insurance, C-section	Yes
Draganov, 2005 [21]	23; JoRRP	PCR, RT-qPCR	HPV11	Yes
Buchinisky, 2008 [30]	118; JoRRP	PCR, RFLP	Early age at onset, HPV11	Yes
Omland, 2014 [4]	221; JoRRP + AoRRP	PCR	HPV negative in AoRRP	No
Tjon Pian Gi, 2015 [23]	55; JoRRP + AoRRP	PCR	Early age of onset, HPV11 in JoRRP, HPV6 in AoRRP	In JoRRP only
Buckinisky, 2019 [32]	339; JoRRP + AoRRP	PCR, RFLP, linear array	Early age at onset	No
Nogueira, 2021 [24]	15 JoRRP, 26 AoRRP	Sequencing	Early age at onset, HPV11 in JoRRP	Yes

* Number of recruited patients tested for HPV strains specified in parenthesis if different than recruited cohort size; ** PCR = polymerase chain reaction, RFLP = restriction fragment length polymorphism, linear array = Roche Linear Array HPV genotyping test; ^1^ HPV6 subtype c is now believed to be HPV11; ^2^ HPV11 and HPV16 were grouped together and not analyzed separately.

**Table 2:** Characteristics of the prospective cohort at the time of recruitment.

Patient ID	Gender	Race ^1^	Age at Onset	Years since Diagnosis	Derkay Score	Prior Surgeries
Pt. 01	Male	White	4	9	12	22
Pt. 02	Male	White	2	6	5	10
Pt. 03	Male	AA	2	8	1	10
Pt. 04	Female	White	2	7	9	7
Pt. 05	Male	White	1	18	11	125
Pt. 06	Female	White	2	18	4	23
Pt. 07	Male	White	7	3	6	0
Pt. 08	Male	AA	7	3	5	0
Pt. 09	Female	White	4	7	19	24
Pt. 10	Male	White	6	5	1	10
Pt. 11	Male	White	6	2	11	0
Pt. 12	Male	White	2	5	8	23
Pt. 13	Male	White	2	7	7	27
Pt. 14	Female	AA	1	10	9	23
Pt. 15	Male	White	1	13	11	108
Pt. 16	Male	Hispanic	7	7	0	14
Pt. 17	Female	White	5	9	5	25
Pt. 18	Female	AA	8	6	7	2
Pt. 19	Male	White	10	7	14	7
Pt. 20	Male	AA	4	2	15	0

^1^ AA = African American.

**Table 3:** Outcomes calculated at end of the study period.

Patient ID	Surgeries in First Year of Recruitment	Total Surgeries 1 y Post- Recruitment	Lifetime Surgeries at End of Study	Surgeries per Year Since Diagnosis	HPV Serotype ^1^
Pt. 01	5	27	32	4	HPV6
Pt. 02	3	13	27	4	HPV6
Pt. 03	4	14	22	3	HPV6
Pt. 04	2	9	17	2	HPV6
Pt. 05	6	131	151	8	HPV11
Pt. 06	6	29	33	2	HPV6
Pt. 07	6	6	9	3	HPV6
Pt. 08	3	3	3	1	HPV6
Pt. 09	7	31	34	5	HPV6
Pt. 10	1	11	11	2	-
Pt. 11	8	8	10	5	HPV6
Pt. 12	8	31	34	7	HPV6
Pt. 13	5	32	34	5	HPV6
Pt. 14	3	26	27	3	HPV6
Pt. 15	6	114	117	9	HPV11
Pt. 16	1	15	15	2	-
Pt. 17	3	28	28	3	-
Pt. 18	1	3	3	1	-
Pt. 19	2	9	9	1	HPV6
Pt. 20	8	8	9	5	HPV6

^1^ Insufficient tissue obtained from surgeries for patients 10, 16, 17, 18 for HPV genotyping.

**Table 4:** Summary of average values for clinical course categories.

Characteristic	Few Surgical Interventions	Regression	Sustained Disease	Progression	Kruskal-Wallis Test ^1^
Age at onset	6 ± 3	5 ± 2	3 ± 2	1	0.04
Years since diagnosis	7 ± 2	6 ± 3	7 ± 5	18	0.54
Derkay scoreat recruitment	6 ± 5	8 ± 3	9 ± 6	11	0.72
Surgeries prior to recruitment	14 ± 9	7 ± 10	25 ± 33	125	0.12
Surgeries in first year after recruitment	2 ± 1	4 ± 2	6 ± 2	6	0.58
Total surgeries 1y post-recruitment	15 ± 10	11 ± 11	31 ± 33	131	0.06
Lifetime surgeries at end of study	16 ± 10	15 ± 13	36 ± 32	151	0.04
Surgeries per year since diagnosis	2 ± 1	3 ± 1	5 ± 2	8	0.02

^1^ Red indicates the statistical significance of *p* < 0.05.

## Data Availability

The published article includes all datasets generated or analyzed during this study.
